# Predictors of Outcomes in Autism Early Intervention: Why Don’t We Know More?

**DOI:** 10.3389/fped.2014.00058

**Published:** 2014-06-20

**Authors:** Giacomo Vivanti, Margot Prior, Katrina Williams, Cheryl Dissanayake

**Affiliations:** ^1^Olga Tennison Autism Research Centre, School of Psychological Science, La Trobe University, Melbourne, VIC, Australia; ^2^Victorian Autism Specific Early Learning and Care Centre, La Trobe University, Melbourne, VIC, Australia; ^3^School of Psychological Sciences, University of Melbourne, Melbourne, VIC, Australia; ^4^Department of Pediatrics, University of Melbourne, Melbourne, VIC, Australia

**Keywords:** autism, early intervention, outcomes, predictors, individual differences

## Abstract

Response to early intervention programs in autism is variable. However, the factors associated with positive versus poor treatment outcomes remain unknown. Hence the issue of which intervention/s should be chosen for an individual child remains a common dilemma. We argue that lack of knowledge on “what works for whom and why” in autism reflects a number of issues in current approaches to outcomes research, and we provide recommendations to address these limitations. These include: a theory-driven selection of putative predictors; the inclusion of proximal measures that are directly relevant to the learning mechanisms demanded by the specific educational strategies; the consideration of family characteristics. Moreover, all data on associations between predictor and outcome variables should be reported in treatment studies.

With increasing advances in autism research over the past decades, it has become clear that clinical heterogeneity is one of the most significant features of autism spectrum disorder (ASD) as diagnosed today. Genetic research indicates that hundreds of genes are implicated in ASD (1); neuropsychological research suggests that multiple neurocognitive mechanisms, rather than a single impairment, might underlie ASD symptoms (2); clinical research points to remarkable heterogeneity at the behavioral/phenotypic level (3, 4). Concomitantly, substantial individual differences are apparent with regard to treatment outcomes in this population (5).

On the basis of group-level data, research suggests that behavioral programs that are implemented as early as possible and in an intensive manner [often referred as early intensive behavioral interventions (EIBI)] can be efficacious in improving cognitive, adaptive, and social–communicative outcomes in young children with ASD (6, 7). However, analysis of treatment response at the individual level indicates that whilst some children show dramatic improvements, some show moderate gains, and others only show minimal or no treatment gains (8). Moreover, there is marked variation in the type and magnitude of positive outcomes seen between studies assessing effectiveness of the same intervention (5, 9–13).

Current knowledge of the factors associated with such individual differences in response to early intervention is limited. Therefore, the positive impact of these programs is hampered by lack of knowledge on the critical issue of “which children benefit from which program” (14). In the absence of this crucial information, the issue of which interventions should be chosen for a particular child is a common dilemma facing both families and clinicians. This question needs to be answered so the most effective early interventions can be provided for every child and to allow service and policy level decision making that will make best use of resources.

Recent literature indicates that choice of early intervention and education programs by families and clinicians is currently linked to factors such as regional proximity to services, anecdotal reports on effectiveness, or persuasive sales pitches/marketing, rather than based on the knowledge of which specific service is likely to result in best outcomes given the individual characteristics of the child (15, 16). This gap in knowledge creates the risk of enrolling children in programs from which they will not gain benefit, leading to a profound emotional and economic burden for affected children, their families, and the community. Given the tremendous implications of this issue at the personal, family, and societal level, in our opinion the identification of predictors of treatment outcomes should be at the top of the autism research agenda. However, advances in this area require a different lens for assessing individual differences as well as the adoption of more fine-grained research methodology, including new theoretical frameworks to analyze profiles of “responders” and “non-responders” under different types of early intervention.

In this paper, we shall argue that information about predictors of treatment response is limited because the current theoretical and methodological approaches are not adequate to address this issue. Furthermore, we will offer some directions for research, and discuss the implications for clinical practice.

## Current Knowledge on Predictors of Early Intervention Outcomes

While there are a number of treatments in the field of ASD, only a small proportion of these have scientific evidence for their efficacy. EIBI programs are currently recommended as the “treatment of choice” for children diagnosed with an ASD (17, 18), with most early intervention research focused on this type of approach. EIBI is characterized by the active engagement of the child for many hours per week (usually 20+) in a planned educational intervention delivered primarily in direct 1:1 child–adult instruction, with specific goals derived from assessment results, manualized/operationalized instructional procedures, and a data collection system to facilitate progress and outcome measurement (11). Within this framework there are different programs, which vary according to the specific curriculum and teaching procedures (19–21).

A number of reviews and meta-analyses have examined group-level outcomes in response to early intervention programs (6, 10, 22), indicating that EIBI programs appear to be effective for increasing adaptive behavior and IQ in young children with ASD. Nevertheless, there are limitations to available research, including lack of randomization in most published studies, small sample sizes, and possible biases due to awareness of treatment status in parents and providers. Moreover, the definitions of EIBI vary across studies, ranging from very specific definitions (e.g., including only programs that strictly adhere to the behavioral procedures originally described by Lovaas) [e.g., Ref. (23)] to very broad ones (including programs that are implemented early and intensively but use teaching procedures that significantly depart from the work of Lovaas, such as Pivotal Response Training) (22).

Importantly, available evidence suggests that EIBI programs (and early educational programs more in general) are not equally beneficial for all treated children (8). In the following, we focus on the factors associated with individual-level variability in response to current early educational intervention approaches, including strictly defined EIBI programs based on the work of Lovaas, as well as programs that are based on behavioral techniques but are not necessarily implemented at a high level of intensity [e.g., Reciprocal Imitation Training (24)], and interventions that meet the intensity requirement but do not have an explicit behavioral orientation [e.g., the PACT program (25)].

Analysis of available evidence indicates that pre-treatment cognitive abilities (IQ) and language abilities are the most often reported correlates of gains in early intervention studies (26–33), although not all studies concur in their conclusions (34–36). Several studies also indicate pre-treatment level of adaptive behaviors as a relevant correlate of treatment gains (6, 37, 38).

Whilst there is some evidence that children who are younger and less severely affected might be more responsive to early intervention, other studies report mixed or negative findings [(31, 39–43); see also Ref. (10, 44)].

A number of studies have identified more specific abilities associated with positive treatment outcomes, including play skills (24, 45–47), interest in objects (48–50), joint attention (46, 47, 50), imitation (38), low social avoidance (51), and response to social versus non-social reinforcement (52). In addition, there have been mixed reports on family factors such as the level of maternal education (53, 54) and family stress (55, 56).

## Limitations with Current Approaches to Research on Predictors of Outcome

Despite such findings on predictors, knowledge of the factors underlying positive treatment outcomes is limited and inconclusive. The major limitations to current research approaches are outlined below.

1. Variables associated with change in treated groups do not necessarily reflect actual predictors of outcomes (i.e., moderators or mediators of treatment response), as not all the observed change can be attributed to treatment (57, 58). If factors other than treatment that might contribute to change are not considered and controlled, correlates of changes within a treated group might or might not tell the full story about prediction of treatment response.
2. The selection of predictor variables is often not theory-driven (59, 60). In most papers reporting on predictors of outcomes, no rationale is provided on why specific variables are selected for analyses. It is apparent that, in most cases, the predictor variables are the measures used to characterize samples at trial commencement, with the purpose of identifying any important differences between randomized groups. Equally, the types of measures used often indicate broad constructs and thus lack the specificity required for a predictor variable. Therefore, the current findings of IQ/language variables as the most consistent predictors of response to early intervention programs tell us more about how commonly these measures are used in baseline assessments in intervention research than about their strength as predictors.
3. Analyses of specific behavioral predictors that could account for individual or subgroup differences are rarely included in research on intervention and prognosis (61). Most intervention studies focus on overall group-level outcome data, and analyses on those factors associated with identifiable subgroups of children or individual differences are either not conducted or not reported, despite the fact that treatment response is so variable in ASD. In medical research, the evaluation of intervention effectiveness often follows a progression from an initial focus on overall group-level outcomes (“does the treatment work for this condition?”) to a subsequent focus on predictors of outcomes for identifiable subgroups or at an individual-level (“what are the factors associated with outcomes?”), particularly when treatment response is variable, of insufficient benefit compared to risk to warrant treatment for some, or when advances are still needed to improve outcomes. In research on autism intervention, most trials are designed to only address the question of group-level efficacy/effectiveness. However, as current research emphasizes the heterogeneity of ASD at every level of analysis, and the results of trials and meta-analyses show variable effect sizes, there is a clear need to move to the next stage of intervention research, with a focus on the specific individual predictors of treatment outcomes, and a focus on moderators and mediators of treatment response.
4. Few studies compare predictors of outcomes across different intervention programs (58). The impact of child characteristics is likely to vary according to the intervention program implemented, so that a child who is not responsive to program X (e.g., discrete trial training) might be more responsive to program Y [e.g., pivotal response training (14, 49)]. Since different programs utilize different instructional techniques (for example, different emphasis on external versus social reinforcements, different emphasis on verbal versus non-verbal instructions), it is likely that children with varying intrinsic cognitive and learning profiles will respond preferentially to different teaching approaches, just as do children who do not have ASD (60, 62, 63). However, the majority of studies on predictors of outcomes include data on response to one program only. Moreover, the inclusion of only one treatment leaves open the possibility that the change predicted by the pre-treatment factors is due to factors other than treatment (59).
5. Intervention studies recruit different samples from those that present at clinical services. Exclusion criteria in many intervention studies result in children with associated medical conditions, seizures, or a low IQs [e.g., IQ < 35 in Ref. (64)], or developmental age [e.g., <12 months in Ref. (25)] being excluded. While these exclusion criteria are set to create homogeneous samples for research purposes, they reduce generalizability to those who are seen in clinical and community-based settings, providing little information about which programs should be recommended based on different pre-treatment characteristics in the wider ASD population. Children with medical conditions, seizures, and/or severe intellectual disability make up a significant proportion of the ASD population (65, 66), and they are more likely to be referred to treatment programs compared to children with no identifiable comorbid features (67, 68). Therefore, research on their responses to different programs is crucial to inform clinical practice, so that enrollment to an intervention service is most likely to offer benefits and cause no harm.
  Current guidelines in clinical trials suggest that excluding patients with associated disorders from research should not occur when these comorbidities are common or when they affect treatment response and prognosis (69). As current research reports an increasing number of comorbid conditions associated with ASDs (66), including dysfunctions that are not diagnosable as specific disorders, the attempt to isolate “pure autism” in the ASD population by excluding “autism complicated by comorbid features” appears to be increasingly impracticable and unrealistic [see Ref. (35)].
6. Standard predictor measures currently used in research are very broad. Omnibus factors such as tested IQ, speech and language assessments, and adaptive behavior have predominated as both predictors and outcome measures in intervention research (53, 60). The use of such broad measures in intervention studies as predictors is problematic for a number of reasons. Low scores in IQ, language, and adaptive behaviors reflect a variety of distinct underlying processes, making it difficult to understand the specific mechanisms underlying the intervention response. Given that performance on IQ tests is, in itself, a measure of learning abilities (e.g., inefficient information processing), it is not surprising that children with lower IQ have more difficulties in learning from educational treatments. To avoid circular reasoning (children who have more difficulties in learning, as measured through pre-treatment IQ testing, are the ones who will have more difficulties in learning from educational treatments), research needs to focus on more proximal predictor variables. These should reflect specific and clearly defined processes that might explain difficulties in responding to educational strategies [e.g., response to social reinforcements in (52); lack of spontaneous imitation in (70)]. Moreover, broad variables such as IQ and communication scores are not robust measures in children younger than 3 years (71), and different tools used to measure IQ might provide different results depending on the instruction formats (e.g., verbal instructions versus demonstration) in the ASD population.
7. Family factors are seldom considered as predictors in outcomes studies. There is very little systematic data on how the characteristics and behaviors of parents influence children’s responses to intervention (72). The motivation of parents to pursue and persist with intervention programs, which involve considerably more effort than administering medications or using complementary and alternative therapies, may be an important factor in treatment outcomes. Moreover, the parents’ personal strengths (e.g., communication style, flexibility) may contribute to the developmental gains of their child. Lack of research in this area is surprising, given that (1) parents are frequently expected to engage as the main therapists for their children in many interventions, even those that are not called parent-mediated interventions, and (2) family factors have been found to impact on treatment response across a number of intervention programs for children with other conditions [see Ref. (73)]. Moreover, family factors such as higher parenting stress, negativity and depression, and low SES are ubiquitous factors in poorer outcomes across a range of child mental health interventions (74–76). The few studies that have investigated family factors in ASD indicate the relevance of parent’s education, responsivity, and beliefs about the importance of child independence (77, 78). Another study (79) reported that higher distress in mothers pre-treatment was associated with lower adaptive behavior outcomes post-treatment, although the effect was not statistically significant [see also Ref. (30)]. Similarly, Osborne et al. (55) reported that high levels of parenting stress reduced the intervention gains in their children, particularly for high intensity interventions. Despite the relevance of this literature, the available evidence on family characteristics moderating treatment outcomes is limited and inconclusive.

## Recommendations

On the basis of the limitations in the intervention outcome literature to date, a number of recommendations are made here.

1. Selection of putative predictors should be theory-driven, and predictors should be proximal and specific rather than broad. In order to match specific learning profiles to specific teaching programs, it is important to conduct a fine-grained analysis of child characteristics. Doing so will enable us to determine not only what the child needs to learn (which will inform treatment objectives) but also how the child learns (which will inform treatment strategies). In addition, we also need to develop a fine-grained understanding of how each treatment works; that is, what are the processes (or the active ingredients) of the intervention that interacts with the child characteristics to promote learning in that child? With time it is likely that an understanding will emerge about the role of different factors on the pathway to effective intervention, such that some will be seen as “first order” or “primary” and required for certain educational or behavioral approaches to be effective, while others will be “second or later order” indicating modification that need to be made to optimize effectiveness of intervention techniques.
  The analysis of the active ingredients of treatment involves a conceptual distinction between moderators of treatment effects, and mediators through which the intervention supposedly works (80). Moderators of treatment outcomes are the pre-treatment characteristics that might determine the degree of effectiveness of treatment versus control, but do not change as a consequence of the intervention, such as chronological age, gender, or maternal education. Conversely, mediators of treatment outcomes are the factors through which a treatment exerts its effects: they are subject to change as a consequence of the treatment, and these changes, in turn, affect treatment outcomes. For example, based on the theoretical tenet and the educational strategies of the Early Start Denver Model (a program in which the therapists are instructed to follow the child’s lead), it is plausible that changes in the spontaneous propensity to initiate social interactions and engage in joint activities mediate outcomes in this program (81). Similarly, it is plausible that changes in the propensity to imitate others mediate outcomes in Reciprocal Imitation Training (24), and that changes in the ability to understand and follow visually mediated task instructions would be relevant in response to the TEACCH program (82).
  The study of mediators of treatment outcomes requires the knowledge of the learning processes upon which the instructional techniques of the teaching program are based, or, in other words, understanding of the active ingredients underlying treatment-related changes. Without such knowledge, selecting among the many variables that are potentially associated with response to treatment in ASD is a difficult task. Importantly, intervention programs should have manualized guidelines and fidelity procedures to ensure that the active ingredients supposedly involved in the therapy are not “diluted” when the programs are translated into community practice.
  A clear definition of the processes through which the child is able to learn in response to the particular instructional techniques is therefore one starting point for defining a specific set of putative moderators and mediators. Furthermore, it is crucial to focus on proximal factors that are known to support learning, with different predictor variables reflecting distinct and defined processes, so that the specific weight of these putative predictors mediating treatment response can be measured. Processes that are known to be foundational for social learning, and might be relevant predictors of outcomes for early educational programs include: social attention [paying attention to people and their actions (83)]; social motivation [which can be operationalized in terms of social approach versus social avoidance behavior, or response to social versus non-social rewards (52, 84)]; intentional communication [using language or non-verbal communication to communicate (85)]; receptive language/communication [understanding others’ communication (86)]; joint attention [both initiation and response to joint attention (87, 88)]; goal understanding (89, 90); imitation (91, 92); functional play (35, 93). All of these processes reflect different facets of social cognition that are known to support social learning, and which are associated with developmental outcomes in ASD as well as in typical development [e.g., Ref. (94, 95)]. Preliminary evidence provides encouragement on the value of these factors in predicting response to intervention [e.g., Ref. (24, 35, 38, 45, 46)]. Another factor that might be associated with outcomes is the extent of restricted/repetitive behaviors (RRBs), since engagement in inflexible routines and insistence on sameness are likely to hinder acquisition of new skills and social learning. For example, Watt et al. (96) reported that prolonged engagement with RBBs was negatively related to social competence across the crucial developmental period from 2 to 3 years. Data on the relevance of RRBs in response to treatment are scant and equivocal (52, 97, 98), so more research is needed to investigate how individual differences in the extent of RBRs affect response to intervention.
  Other factors that are not specific to ASD might also be associated with treatment outcomes across intervention programs. These include attention (in particular sustained attention, e.g., the ability to be focused on a task from the beginning to the end); memory; responsivity to instrumental learning/conditioning (the ability to associate contingent rewards to own behaviors); processing speed and efficiency; generalization (the ability to use what has been learned in non-training situations). Rate of learning (e.g., number of treatment goals achieved in the first 6 months of treatment) might also be a relevant predictor of outcomes (99). Research on the predictive value of these factors in response to treatment is scant.
  As standardized tests are not available for many of the processes listed above, it is necessary to develop and utilize novel, fine-grained experimental measures and observational protocols that are suitable for young children with ASD across the spectrum of severity [e.g., Ref. (35, 52)]. Table 1 summarizes a list of the factors that have been theoretically and/or empirically linked to positive treatment outcomes (including both child and family factors, discussed in the next section), and for which psychometric development is needed to advance a methodology for identifying predictors of outcomes.
2. Family factors should be investigated in treatment studies. As family involvement is a recommended component of early intervention (100), future research should systematically investigate the family characteristics associated with responses to treatment for children with ASD. Importantly, different early intervention programs involve instructional techniques (e.g., play-based versus highly structured strategies) that may or may not fit with a family’s educational practice and cultural values. Different families might also respond differently to the parent training formats used by the different programs, which vary from information sessions in small groups to 1:1 sessions in which the parent is asked to implement educational strategies under a therapist’s supervision. Other potential family factors mediating response to treatment are the level of parent/therapist collaboration, maintaining positive expectations for child outcomes, lack of family conflict, positive father involvement, level of social support, and level of stress in the family (14).
  High levels of child behavior problems are prominent in families with children with developmental disabilities and contribute to greater stress in families than cognitive delay (101, 102). Generally, mothers of children with ASD report higher levels of distress than those of non-disabled children or those with children with other disabilities, although there is considerable variability across families (103, 104). In a recent paper, Benson (105) has shown that social network attributes, including the range and function of emotional support, are related to perceived social support, which in turn can bring about a decrease in depressed mood in mothers of children with ASD. Formal and informal social support networks can have the effect of enhancing quality of life, confidence in parenting and optimism (106) for families with children with disabilities, and might be crucial factors predicting treatment outcomes.
  Accurate measures that appropriately capture individual differences between families in terms of specific values, attitudes, and resources may need to be developed. Future research should investigate the role of family factors in a systematic way, selecting specific, theory-driven variables, developing novel fine-grained measures and comparing variables associated to outcomes across different programs. When examining a broad array of putative factors (e.g., family characteristics and attitudes, sociodemographic background) that may be associated with outcomes, it is important to consider that while a single factor might not be predictive of outcomes, the accumulation of multiple risks often is. Studies looking at longitudinal outcomes in at-risk populations often use cumulative risk approaches (107) in which a discreet number of risk indexes are created to capture the level of risk within a number of predefined theoretically driven domains. To illustrate, a number of studies [e.g., Ref. (108, 109)] have examined the predictive value of risk factors within the child (various child attributes), sociocultural (demographic characteristics), and parenting domains (parent beliefs/attitudes), showing that cumulative risk within each domain predicted outcomes in typically developing children. This approach can be helpful in examining whether risk factors in family related and sociodemographic domains play a role in response to treatment in the ASD population.
3. Studies on treatment efficacy/effectiveness should always report on individual differences in response to treatment as well as those factors associated with positive treatment outcomes. The analysis of individual differences (i.e., characteristics of responders and non-responders) should become a standard procedure in early intervention research [similarly to current procedures in pharmacological intervention research (110)]. Moreover, when predictor variables are measured, their association with the outcome measures should always be reported. The tendency to report on predictors of outcomes only when results indicate significant associations makes it hard to draw clear conclusions on the robustness and consistency of factors associated with treatment response. Thus, it should be mandated that all data on predictor and outcome variables are reported, as in the case of outcome measures in clinical trials (111). Reporting all available information on the association between predictors and outcomes can also be useful in studies with small sample sizes, which comprise the majority in the field of ASD, so that data from multiple small trials can be pooled for meta-analyses. While data on correlates of treatment gains *per se* do not allow for definite conclusions on predictors of treatment outcomes, this information can be critical to inform subsequent research designed to control for the predictive value of factors that appear to be relevant in relation to treatment changes.
4. Research on predictors of treatment outcomes should compare responses across different programs. As mentioned above, the analysis of correlates of gains in a treated group might not be informative on predictors of treatment outcomes, as not all observed change in a treatment study can be attributed to the treatment. Research on predictors of treatment outcomes should use designs that compare different programs, thus allowing for the analysis of the interaction between selected participant characteristics X treatment group. This approach is critical not only from a methodological point of view (distinguishing moderated treatment effects from factors that are associated with outcomes/changes in ASD more generally), but also from a clinical perspective. Different early intervention programs, while sharing many similarities, involve distinct instructional techniques that are based on different theories and which tap into different learning processes. For example, some programs involve an emphasis on teaching words/sentences that have the instrumental function of obtaining desired items, whilst in other programs language is targeted with an emphasis on its social versus instrumental function. Similarly, programs vary across a number of teaching processes (e.g., verbal versus visually conveyed instructions, following the child’s lead versus adult-directed models), reflecting different underlying theoretical frameworks. Since these different teaching practices require different learning processes on the part of the learner, it is possible that different types of learners will respond to different types of teaching procedures. Given that remarkable differences are present both in the teaching procedures of the different programs and in the social, cognitive, and learning profiles of children with ASD, it is imperative that future research compares the profiles of response to different early learning programs that use different instructional techniques. This approach would allow identification of the pre-treatment child characteristics that support learning when a specific set of teaching procedures is used. Only a few studies to date have provided information on the specificity of predictors of outcomes to one versus another treatment program [see Ref. (49, 50)].
5. Research on predictors of outcomes should involve large heterogeneous samples. It is important for studies on the predictors of response to treatment to include the full range/spectrum of children with ASD. Many children with comorbid conditions are typically excluded from research studies, such as those with severe intellectual disability and associated medical conditions or seizures. However, as discussed above, these children represent a substantial proportion of the ASD population referred to community early intervention centers. It is particularly important that research focuses on children with very low IQ to determine whether the currently available early intervention programs are appropriate for this population, and which specific factors are predictive of positive outcomes (e.g., short attention span, passivity, poor play skills). If research shows that there are child characteristics that indicate a lack of responsivity to any of the currently available early intervention programs, we need to develop novel approaches targeting the specific impairments of the “non-responders,” rather than referring them routinely to programs from which they do not benefit.

**Table 1:** **Putative predictors of treatment outcomes that require standardization/psychometric development**.

Child factors	Family factors
Functional communication (requesting, protesting)	Sociodemographic background (resource-poor versus resource-rich)
Social communication (sharing, commenting)	Family expectations about treatment
Social approach (versus avoidance)	Family sense of competence/self-efficacy
Joint attention	
Social understanding	Parent/therapist alliance
Imitation	Family stress and discord
Level of RRBs	Father positive involvement
Functional play with objects	Social support
Responsivity to reward learning	
Generalization	
Core neuropsychological functions (processing speed/efficiency, sustained attention, cognitive control, memory)	

## Conclusion

Little is known about predictors, moderators, and mediators of treatment response in children with neurodevelopmental and neuropsychiatric conditions (112, 113), and the field of ASD is no exception. With recent advances in research documenting the positive impact of early intensive behavioral programs for children with ASD, the critical issue facing researchers, clinicians, and practitioners in the field is not as much a lack of evidence-based treatments, but rather an inability to predict which treatment will work best for each child. The variability of response to EIBI and other early intervention programs is a phenomenon that very likely reflects the heterogeneity of the ASD population. While some crude prognostic variables, such as IQ level, hold some value in predicting which children will respond best to early intervention, to date, the available information on moderators and mediators of treatment response is inconclusive. In this paper, we argue that lack of knowledge on “what works for whom and why” in ASD reflects a number of methodological and theoretical issues in ASD treatment research.

**Figure 1:**
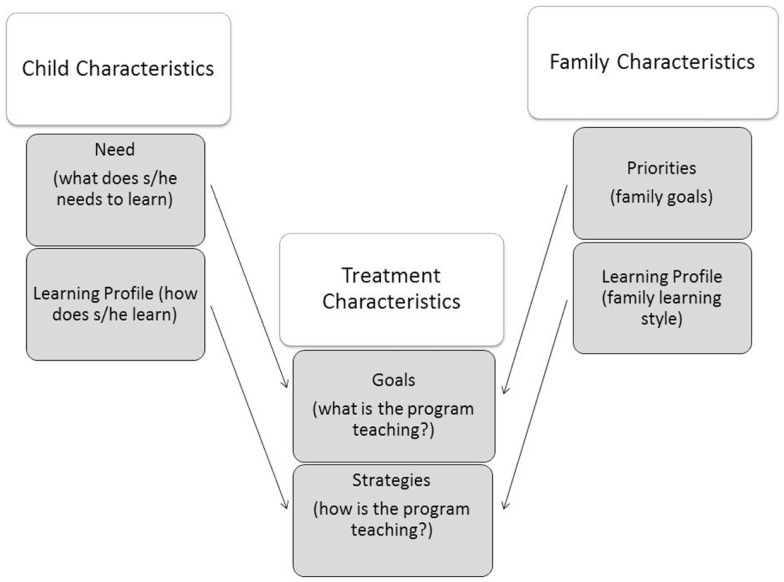
**Treatment goals should be selected on the basis of the child’s needs and family priorities**. Treatment strategies should be selected on the basis of the child learning profile and the family’s learning style.

We argue that the selection of treatment objectives and treatment strategies should be informed by knowledge of: (1) child characteristics (how does the child learn best, and what does s/he needs to learn); (2) family characteristics (what are the family priorities/expectations, and what is the family learning style); (3) treatment characteristics (what are the treatment aims, and how does the treatment work). This model is illustrated in Figure 1. Current understanding on the interplay of these factors in determining treatment outcomes is limited, and we have offered here a number of recommendations to advance knowledge in the field. Given the complexity of the biological underpinnings of ASD, behavioral predictors might not be sufficient to predict treatment outcomes to a high degree of accuracy. Nevertheless, knowledge arising from this line of research can critically contribute to the development of decision-making guidelines in the field of ASD early intervention.

A number of questions remain, which need further critical evaluation. First, it is unclear whether predictors of intervention outcomes are specific to ASD. As reported above, a number of specific factors in the two areas of impairments that characterize ASD (social communication and repetitive behaviors domains) are likely to hinder positive responses to intervention; however, other factors that are not specific to ASD (e.g., attention span, memory) might also play an important role in predicting outcomes. For example, IQ and language abilities are associated with outcomes across a number of conditions [e.g., schizophrenia (114), depression (115), conduct disorder (116)] that are not related to ASD. Indeed, these factors might predict outcomes above and beyond receiving early intervention *per se* [see Ref. (117–119)].

It is possible that factors associated with positive responses to intervention are the same that support positive learning in children without an ASD. More research is needed, therefore, to achieve a more fine-grained understanding of mechanisms supporting early learning and cognitive development in typical development as well as atypical development (e.g., neuropsychological processes such as attention and processing speed and social processes such as social engagement, sharing of affect, and joint attention). Sophisticated research designs are also needed to identify distinctions and overlaps and the interplay between variables moderating treatment changes, those mediating outcomes, and those affecting both processes.

Given the scarcity of standardized tools to assess social and non-social factors that might be relevant for response to intervention, measurement of putative predictors of outcomes poses relevant challenges. More efforts are currently needed to develop systematic observational and experimental tasks that provide reliable measurements of such predictors.

Another critical aspect concerns the definition of positive versus nil or minimal outcomes in ASD research. Outcome measures used in treatment research might not be sufficiently sensitive to capture gains in children making very slow or very small gains. However, these small progressions might have a relevant impact on the family and child’s quality of life and may be the necessary “first order” changes that are needed to allow further positive response. Thus, the operational definition of “responders” versus “non-responders” should take into account different outcome criteria besides the standard/conventional measures used in ASD research.

Moreover intervention in ASD, while promoting gains in certain areas, can also be instrumental in preventing declines or worsening in other domains. For example, there is evidence that some repetitive behaviors might increase over time in ASD (120), and it is conceivable that a treatment program could aim to prevent such increase. In this case, the absence of change in repetitive behaviors in children undergoing treatment cannot be seen as evidence of a lack of treatment response. Therefore, definitions of responders and non-responders must be conceptualized and framed on knowledge of developmental patterns in the different treatment target domains.

A related issue is the question of when do we begin to classify children as “non-responders”? If children are not showing measurable gains after 1 year, is it possible that they will start responding in the second year? Currently, our knowledge on the timing of treatment response is limited. Different studies provide information on predictors of outcomes in relation to programs that vary in duration, making it difficult to compare results and to calibrate expectations about the timing of treatment response. In order for research to inform clinical decision-making, some consensus about the timing of expected response to treatment is therefore necessary, and approaches that allow time-limited intervention with ongoing follow-up to allow future intervention planning should be developed.

A further challenge is the clinical management of “non-responders” to early intervention programs. In the best-case scenario, future research will indicate that those children who do not respond to some early intervention programs will nonetheless respond to other available programs, so that each child can be matched to the most appropriate program. However, it is possible that specific child or family factors are associated with minimal or no responses across all available treatment options. If this is the case, it will be necessary to target the specific factors that are known to limit the efficacy of the program [“treating the constraints” approach (59)], and to conduct specific research focused on these “non-responders.” Until research indicates successful strategies to address the factors limiting response to treatment in this subgroup, the question is: should children who present with a “non-responder” profile be referred to programs from which they will not benefit? This raises a number of ethical issues as well as concerns with regard to cost–benefits considerations, and individual rights to access services.

Finally, the research recommendations that we have outlined, which involve a focus on individual differences in large heterogeneous samples, the measurement of a variety of theory-driven predictor variables at baseline, and the comparison of prognostic indicators across different programs, are expensive ones. To have a sufficient number of participants to identify robust profiles of responders and non-responders to available early intervention programs and to replicate findings across sites requires large-scale collaborative multisite research. Nonetheless, the practical implications of such a research program surely justify the necessary investment, especially as ASD is currently diagnosed in more than 1 in every 100 children and the problems associated with ASD have a high impact for the individual, their family, and the community.

## Conflict of Interest Statement

The authors declare that the research was conducted in the absence of any commercial or financial relationships that could be construed as a potential conflict of interest.
